# Optimization of protein production by *Micrococcus luteus* for exploring pollutant-degrading uncultured bacteria

**DOI:** 10.1186/2193-1801-3-117

**Published:** 2014-02-28

**Authors:** Xiaomei Su, Yindong Liu, Jinxing Hu, Linxian Ding, Chaofeng Shen

**Affiliations:** Department of Environmental Engineering, College of Environmental and Resource Sciences, Zhejiang University, Yuhangtang Road 866#, Hangzhou, 310058 China; College of Geography and Environmental Science, Zhejiang Normal University, Jinhua, 321004 China

**Keywords:** Unculture bacteria, Protein production, *Micrococcus luteus*, Resuscitation-promoting factor, Response surface methodology

## Abstract

The screening of pollutant-degrading bacteria are limited due to most of bacteria in the natural environment cannot be cultivated. For the purpose of resuscitating and stimulating “viable but non-culturable” (VBNC) or uncultured bacteria, *Micrococcus luteus* proteins are more convenient and cost-effective than purified resuscitation-promoting factor (Rpf) protein. In this study, medium composition and culture conditions were optimized by using statistical experimental design and analysis to enhance protein production by *M. luteus*. The most important variables influencing protein production were determined using the Plackett-Burman design (PBD) and then central composite design (CCD) was adopted to optimize medium composition and culture conditions to achieve maximum protein yield. Results showed that the maximum protein yield of 25.13 mg/L (*vs.* 25.66 mg/L predicted) was obtained when the mineral solution, Lithium L-lactate, initial pH and incubation time were set at 1.5 ml/L, 8.75 g/L, 7.5 and 48 h, respectively. The predicated values calculated with the model were very close to the experimental values. Protein production was obviously increased with optimization fitting well with the observed fluorescence intensity. These results verified the feasibility and accuracy of this optimization strategy. This study provides promising information for exploring highly desirable pollutant-degrading microorganisms.

## Introduction

To date, just over 7000 well-founded species of bacteria have been described (Epstein 2013), because most of bacteria in the natural environment cannot be cultivated. It is very common for bacteria to survive under extreme conditions by entering into a “viable but non-culturable” (VBNC) state, where the cells are intact and alive and can resuscitate when surrounding conditions are more favorable (Oliver 2005; Oliver 2010; Pawlowski et al. 2011). Although these VBNC or uncultured bacteria can be studied by molecular ecology methods (Vartoukian et al. 2010), to elucidate their related function and genotype, it is necessary to isolate these bacteria and study their microbiology in pure cultures (Kaeberlein et al. 2002). Meanwhile, the study of VBNC or uncultured bacteria may help explain the nature of microbial uncultivability, and explore the industrial and environmental significance of as-yet-uncultured bacteria.

The most exciting development in resuscitating VBNC bacteria was the discovery of a bacterial cytokine, namely, resuscitation-promoting factor (Rpf), secreted by *Micrococcus luteus* (Mukamolova et al. 1998). Rpf (activity at picomolar concentrations) could promote the resuscitation and growth of high G + C Gram-positive organisms, including *Mycobacterium*, *Rhodococcus*, *Arthrobacter*, *Leifsonia, Bacillus*, *Nocardia*, *Kitasatospora* and *Streptomyces* (Su et al. 2013)*.* At present, more than 30 genes from various microorganisms coded for “Rpf-like” proteins were grouped into Rpf family (Telkov et al. 2006). Specially, the high-GC Gram-positive bacteria (Actinomycetales), of a family of proteins that act as autocrine growth factors (cytokines) were mainly investigated (Kell and Young 2000). Despite many studies on Rpf family proteins and their function in resuscitating VBNC bacteria and stimulating the growth of bacteria (Mukamolova et al. 2002; Panutdaporn et al. 2006; Su et al. 2013), the mechanism of action remains unclear.

Telkov et al. (2006) indicated that Rpf was a peptidoglycan-hydrolyzing enzyme, and strongly suggested that this specific activity was responsible for its growth promotion and resuscitation activity. Moreover, Mukamolova et al. (2006) demonstrated that Rpf stimulated bacterial culturability and resuscitation due to its muralytic activity. However, pure Rpf protein, both native (purified from *M. luteus* culture supernatant) and recombinant was prone to lose its activity after storage at 4°C for 1 week. Recombinant Rpf protein was also less active than native Rpf protein. Furthermore, in the *M. luteus* culture supernatant, several other proteins had been found to possess the same muralytic activity as Rpf protein (Mukamolova et al. 2006). The resuscitation and stimulatory activities of proteins from *M. luteus* culture supernatant had been recently verified (Ding 2004; Su et al. 2013; Su et al. 2013). Therefore, for the purpose of resuscitating and stimulating VBNC or uncultured bacteria, proteins from *M. luteus* culture supernatant are more convenient and cost-effective than purified Rpf protein. While some studies have focused on the function of Rpf protein from the perspective of medicine and epidemiology (Dwivedi and Jaykus 2011; Hett and Rubin 2008), little has been done to investigate the capability of proteins from *M. luteus* culture supernatant to aid in culturing difficult-to-culture bacteria, and for exploring potential environmental functions of VBNC or uncultured bacteria.

It is of great significance to use proteins from *M. luteus* for isolating and culturing highly desirable pollutant-degrading microorganisms, in which case the optimization of medium composition and culture conditions for protein production are very important. To our knowledge, limited information is currently available regarding the optimization of protein production from *M. luteus*. Conventional methods for multifactor optimization are laborious, time-consuming and cannot predict the true optimum due to interactions between the factors (Giordano et al. 2013; Vasilev et al. 2013). Response surface methodology (RSM) based on factorial design and regression analysis overcomes the limitations of single-factor optimization and is more applicable to multivariable systems (Dash and Gummadi 2007). Although some information may be lost or difficult to interpret in fractional factorial designs, it has been widely applied for product and process improvement (Singh et al. 2013; Song et al. 2012).

The aim of the present study was to optimize the medium composition and culture conditions by RSM. Plackett-Burman design (PBD) was used as the first step to screen for the significant variables. Central composite design (CCD) and response surface analysis were then used to optimize the levels of the screened variables that significantly influenced protein production by *M. luteus*.

## Results and discussion

### Evaluation of significant variables affecting protein production

PBD was applied to determine the relative significance of seven medium components and four culture conditions. The effect of each variable on protein production was estimated by the difference between the average of measurements made at the high level (+1) and the low level (-1) of the particular factor. As shown in Table 1, the protein yield varied from 11.84 to 30.25 mg/L. Using the regression analysis in Table 2, the mineral solution, Lithium L-lactate, initial pH and incubation time were identified as significant variables affecting protein production.

**Table 1:** **Plackett-Burman design matrix for 11 variables with coded values along with observed results**

Run	Code variable level											Protein yield (mg/L)
	X_1_	X_2_	X_3_	X_4_	X_5_	X_6_	X_7_	X_8_	X_9_	X_10_	X_11_	
1	+1	-1	-1	-1	-1	+1	-1	+1	-1	-1	+1	13.27 ± 0.26
2	-1	+1	+1	-1	+1	+1	-1	-1	-1	-1	+1	13.94 ± 1.57
3	+1	+1	-1	+1	+1	-1	-1	-1	-1	+1	-1	16.42 ± 0.63
4	+1	-1	+1	+1	-1	-1	-1	-1	+1	-1	+1	16.74 ± 0.66
5	-1	+1	+1	-1	-1	-1	-1	+1	-1	+1	-1	11.84 ± 0.08
6	-1	+1	-1	+1	-1	+1	+1	+1	+1	-1	-1	21.60 ± 0.85
7	+1	+1	+1	+1	-1	-1	+1	+1	-1	+1	+1	20.05 ± 0.81
8	+1	+1	-1	-1	+1	+1	-1	+1	+1	-1	-1	29.17 ± 0.47
9	-1	+1	+1	+1	+1	-1	-1	+1	+1	-1	+1	24.56 ± 0.55
10	+1	-1	-1	+1	+1	-1	+1	+1	-1	-1	-1	18.89 ± 1.44
11	-1	+1	-1	+1	+1	+1	+1	-1	-1	+1	+1	17.70 ± 1.12
12	-1	-1	+1	-1	+1	-1	+1	+1	+1	+1	-1	13.19 ± 1.43
13	+1	-1	+1	-1	+1	+1	+1	+1	-1	-1	+1	13.72 ± 2.08
14	+1	+1	-1	-1	-1	-1	+1	-1	+1	-1	+1	20.21 ± 0.67
15	-1	-1	-1	+1	-1	+1	-1	+1	+1	+1	+1	30.25 ± 0.98
16	-1	-1	+1	+1	-1	+1	+1	-1	-1	-1	-1	17.86 ± 1.36
17	+1	-1	+1	+1	+1	+1	-1	-1	+1	+1	-1	14.50 ± 1.11
18	+1	+1	+1	-1	-1	+1	+1	-1	+1	+1	-1	13.94 ± 0.78
19	-1	-1	-1	-1	+1	-1	+1	-1	+1	+1	+1	16.97 ± 0.43
20	-1	-1	-1	-1	-1	-1	-1	-1	-1	-1	-1	15.31 ± 0.32

**Table 2:** **Variables and test levels for Plackett-Burman experiment (** ^**a**^ **indicates model terms are significant)**

Code	Variable	Low lever	High lever	Protein yield (mg/L)	
		(-1)	(+1)	***t***-value	***P***-value
X_1_	NH_4_Cl (g/L)	2	4	-0.475	0.637
X_2_	KH_2_PO_4_ (g/L)	1.4	2.8	1.433	0.157
X_3_	Mineral solution (ml/L)	1	2	-3.215	0.002^a^
X_4_	Lithium L-lactate (g/L)	5	10	2.987	0.004^a^
X_5_	MgSO_4_ (g/L)	0.03	0.06	-0.151	0.88
X_6_	L-Methionine (g/L)	0.02	0.04	0.89	0.378
X_7_	Inosine (g/L)	1	2	-0.899	0.374
X_8_	Initial pH	7.5	9	2.617	0.013^a^
X_9_	Incubation time (h)	48	96	3.48	0.001^a^
X_10_	Incubation temperature (°C)	30	37	-1.097	0.277
X_11_	Inculum size (%)	2	4	1.116	0.269

### Optimization of the significant variables for protein production

The optimal levels for the significant variables and the effect of their interactions on protein production were further investigated by the CCD of RSM. The design matrix and the corresponding experimental results were shown in Table 3. By applying multiple regression analysis to the experimental data, the correlation between significant variables and protein production was established by the following second-order polynomial equation:1

where Y was the predicted protein yield, X_3_, X_4_, X_8_ and X_9_ were the actual values of mineral solution, Lithium L-lactate, initial pH and incubation time, respectively.

**Table 3:** **Central composite design for optimizing significant variables for protein production**

Run	Mineral solution (ml/L)	Lithium L-lactate (g/L)	Initial pH	Incubation time (h)	Protein yield (mg/L)	
					Actual value	Predicted value
1	1.5	7.5	8.25	72	20.92 ± 0.45	20.84
2	2	5	7.5	48	22.94 ± 0.23	22.4
3	1.5	7.5	8.25	120	1.53 ± 0.33	1.88
4	1	5	7.5	48	21.21 ± 0.12	20.16
5	2	5	7.5	96	13.93 ± 0.38	12.01
6	2	5	9	48	13.11 ± 0.66	13.32
7	2	10	9	96	6.71 ± 0.49	7.31
8	1.5	7.5	9.75	72	7.81 ± 0.61	7.51
9	1	5	7.5	96	9.22 ± 0.36	9.25
10	1.5	2.5	8.25	72	11.71 ± 0.76	13.05
11	2	10	9	48	17.01 ± 0.43	15.59
12	1	5	9	96	4.70 ± 0.56	6.38
13	1.5	7.5	8.25	72	20.31 ± 0.57	20.84
14	1	10	7.5	96	12.08 ± 0.30	11.41
15	1.5	7.5	8.25	72	21.03 ± 0.49	20.84
16	1.5	7.5	8.25	72	21.18 ± 1.16	20.84
17	1	10	9	96	9.00 ± 0.36	8.16
18	1	10	9	48	15.50 ± 0.89	16.97
19	1.5	7.5	8.25	24	19.58 ± 0.54	21.08
20	1.5	7.5	8.25	72	20.97 ± 0.43	20.84
21	2	5	9	96	7.32 ± 0.53	5.84
22	2	10	7.5	48	27.19 ± 0.35	25.05
23	1	10	7.5	48	23.02 ± 0.34	23.12
24	0.5	7.5	8.25	72	15.74 ± 0.34	15.19
25	2.5	7.5	8.25	72	14.17 ± 0.60	16.57
26	1.5	7.5	8.25	72	20.61 ± 0.83	20.84
27	2	10	7.5	96	13.79 ± 0.74	13.86
28	1	5	9	48	15.83 ± 1.44	14.38
29	1.5	7.5	6.75	72	17.69 ± 0.50	19.84
30	1.5	12.5	8.25	72	16.98 ± 0.31	17.48

Analysis of variance (ANOVA) and *F*-test were conducted to evaluate the statistical significance of the fit for Eq. (1) using the Design Expert Software. As shown in Table 4, the Model *F*-value was 30.11, implying the model was highly significant and there was only lower than 0.01% chance that the “Model *F*-value” could occur due to noise. The significance of each variable and their interactions were evaluated by the *P*-values, which indicated that X_4_, X_8_, X_9_, , ,  and  were significant model terms. Additionally, the model fit was verified by a high determination coefficient (R^2^ = 0.966), which indicated that 96.6% of the response variability could be explained. The predicted determination coefficient (Predicted R^2^ = 0.804) was in reasonable agreement with the adjust determination coefficient (Adjusted R^2^ = 0.934), which also confirmed the significance of the model.

**Table 4:** **ANOVA for response surface quadratic model for protein production**

Factors	Statistics				
	Sum of squares	df	Mean square	***F***-value	***P***-value
Model	1075.47	14	76.82	30.11	< 0.0001
X_3_	2.88	1	2.88	1.13	0.3051
X_4_	29.42	1	29.42	11.53	0.0040
X_8_	227.79	1	227.79	89.29	< 0.0001
X_9_	552.46	1	552.46	216.56	< 0.0001
X_3_ X_4_	0.10	1	0.10	0.038	0.8475
X_3_ X_8_	10.90	1	10.90	4.27	0.0565
X_3_ X_9_	0.27	1	0.27	0.10	0.7507
X_4_ X_8_	0.15	1	0.15	0.06	0.8147
X_4_ X_9_	0.64	1	0.645	0.25	0.6225
X_8_ X_9_	8.43	1	8.43	3.30	0.0892
	42.09	1	42.09	16.50	0.0010
	53.19	1	53.19	20.85	0.0004
	87.85	1	87.85	34.44	< 0.0001
	150.01	1	150.01	58.80	< 0.0001
Residual	38.27	15	2.55		
Pure error	0.51	5	0.10		
Cor total	1113.73	29			

Coefficient of determination (R^2^ = 0.966), R^2^(predict) = 0.804, R^2^(adjust) = 0.934.

**Figure 1:**
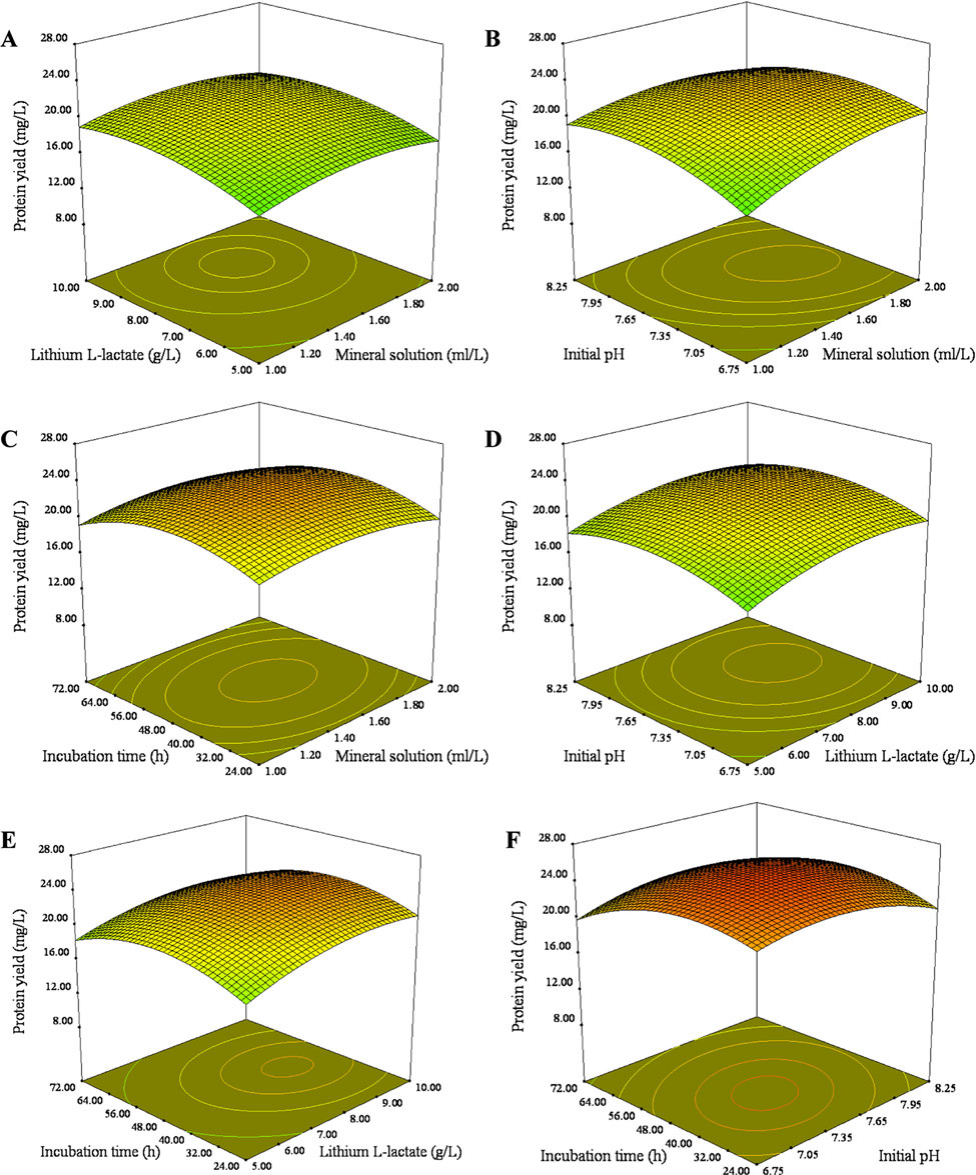
**Response surface plot showing interactive effect of selected variables on protein yield by** ***M. luteus.*** **(A)** Mineral solution and Lithium L-lactate, **(B)** Mineral solution and initial pH, **(C)** Mineral solution and incubation time, **(D)** Lithium L-lactate and initial pH, **(E)** Lithium L-lactate and incubation time, **(F)** Initial pH and incubation time.

**Figure 2:**
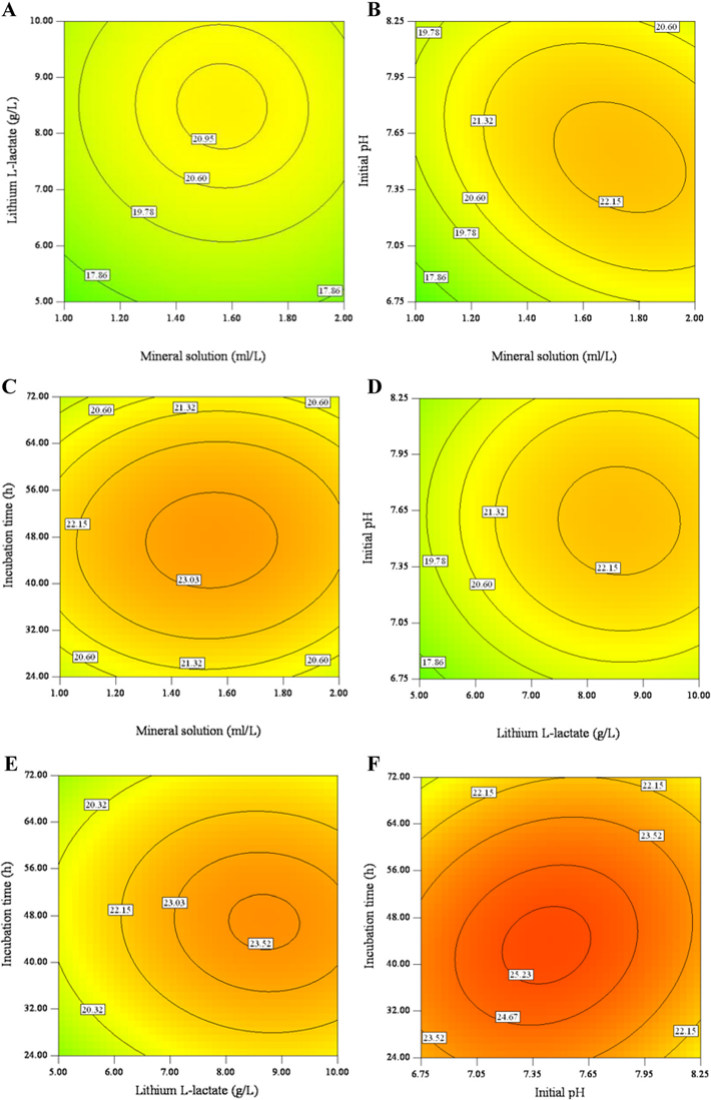
**Contour plot showing interactive effect of selected variables on protein yield by** ***M. luteus.*** **(A)** Mineral solution and Lithium L-lactate, **(B)** Mineral solution and initial pH, **(C)** Mineral solution and incubation time, **(D)** Lithium L-lactate and initial pH, **(E)** Lithium L-lactate and incubation time, **(F)** Initial pH and incubation time.

The 3D response surface plots and the corresponding 2D contour plots that graphically represented the regression equations were described. Figures 1 and 2 demonstrated the relationships between response and experimental levels for each variable. Each figure depicted the effect of two variables while keeping the other variables at their zero levels, and the interaction between two variables could be clearly illustrated (Chen et al. 2013). The response surface in each figure was elliptical and the maximum point was determined from the intersection of major and minor axes of the ellipse. As shown in the Figures 1 and 2, maximum protein production could be obtained when the ranges of mineral solution, Lithium L-lactate, initial pH and incubation time were 1.4-1.8 ml/L, 8-9 g/L, 7.05-7.65 and 40-56 h, respectively. It was important to highlight that the interaction of mineral solution and initial pH had a significant negative effect on protein production while a positive interaction was observed between initial pH and incubation time.

Based on the Eq. (1), the maximum protein production predicted by the Design Expert Software could be achieved when the variables of mineral solution, Lithium L-lactate, initial pH and incubation time were set at 1.5 ml/L, 8.75 g/L, 7.5 and 48 h, respectively. The maximum predicted value of protein yield obtained was 25.66 mg/L.

### Validation of the models and the optimized conditions

In order to confirm the validity of the optimization results, the optimized medium components and culture conditions were evaluated in triplicate which yielded a mean protein value of 25.13 mg/L. The high correlation between the predicted (25.66 mg/L) and actual (25.13 mg/L) values confirmed the model validation. Moreover, the maximum protein yield (25.13 mg/L) obtained after optimization was 24.3% higher than that obtained without optimization. Apparently, the model was confirmed to be accurate and reliable for predicting protein production by *M. luteus*.

**Figure 3:**
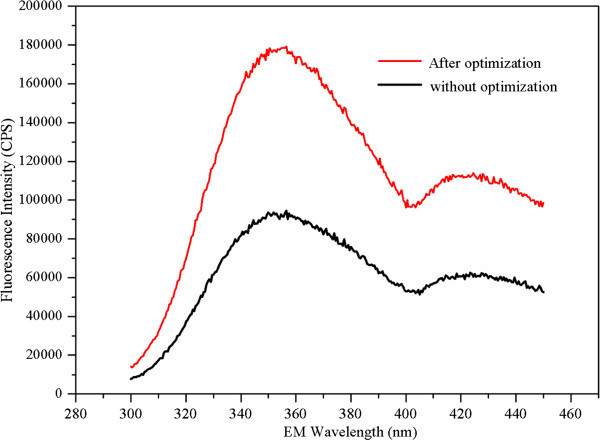
**Emission fluorescence spectra of protein yield in** ***M. luteus*** **culture supernatant with and without optimization.**

**Figure 4:**
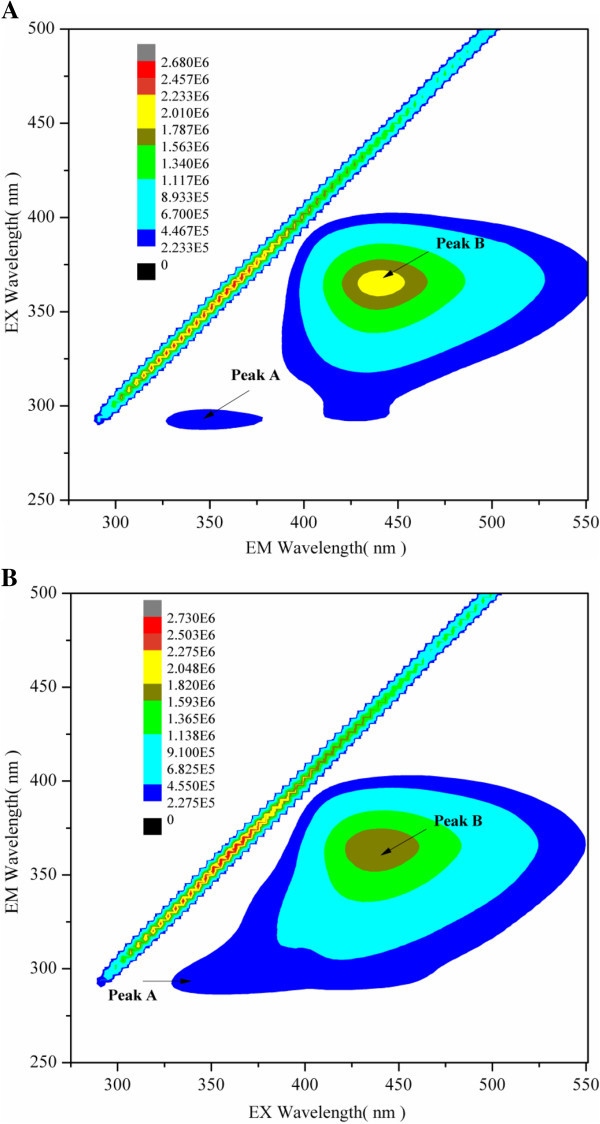
**Fluorescence spectra of culture supernatant from** ***M. luteus*** **with and without optimization. (A)** Without optimization, **(B)** With optimization, Peak A: protein-like substances, Peak B: humic-like substances.

In proteins, the tryptophan is the constituent amino acids with the highest fluorescence emission. And the fluorescence spectrum of a protein is determined by the dominant fluorescent tryptophan (Keeratiurai et al. 2012; Mendonça et al. 2013). Hence, the changes in the tryptophan spectrum were used to indicate the changes of protein concentration. Emission fluorescence spectra of protein concentration in *M. luteus* culture supernatant with and without optimization were shown in Figure 3. It was apparent that the *M. luteus* culture supernatant had maximum fluorescence intensity at 350 nm (excitation at 280nm) which was typical for tryptophan (λ_ex_ 280 nm, λ_em_ 350 nm). It is interesting to point out that a maximum of 2-fold increase in fluorescence was achieved with optimization. In addition, two peaks (peak A and peak B) with relatively high fluorescence intensity could be obviously observed in the three-dimensional fluorescence contour map (Figure 4). As shown in Figure 4, the first main peak was identified at excitation/emission wavelengths (Ex/Em) of 350-400/415-475 nm (peak B), while the second main peak was identified at Ex/Em of 280-290/325-375 nm (peak A). Compared with Figure 4A, the fluorescence intensity of peak A in Figure 4B was significantly increased, while the fluorescence intensity of peak B was decreased. Generally, fluorescence peaks with Em < 380 nm represent protein-like substances, and fluorescence peaks with Em > 380 nm represent humic-like substances (Murphy et al. 2011; Li et al. 2013). In this study, peak B had been described as protein-like peaks, in which the fluorescence is associated with the aromatic amino acid tryptophan. Thus, fluorescence intensity of proteins increased obviously after optimization. In addition, it was worth to note that fluorescence intensity of humic-like substances was much stronger than that of proteins, which demonstrated that protein concentration was relatively low in *M. luteus* culture supernatant. Recently, the resuscitation and stimulatory activities of proteins from *M. luteus* culture supernatant had been widely accepted (Ding 2004; Su et al. 2013; Su et al. 2013). It can thus provide some clues that Rpf at picomolar concentrations could greatly promote the resuscitation and growth of bacteria (Mukamolova et al. 1998; Mukamolova et al. 2006). In addition, the effect of proteins from *M. luteus* on the performance of biphenyl biodegradation and bacterial community in polychlorinated biphenyl (PCB)-contaminated sediments near e-waste recycling sites from Taizhou area (Shen et al. 2007) was investigated. After medium composition and culture conditions optimization, proteins in culture supernatants of *M. luteus* present enhanced activity in resuscitating and stimulating biphenyl-degrading bacteria (data not shown).

At present, increasing concern has surrounded the limitations of current bioremediation which include the poor capabilities of microbial communities in the field (Megharaj et al. 2011). *M. luteus* protein, as an efficient approach to resuscitate and stimulate the VBNC or uncultured bacteria, was a promising method for exploring highly desirable pollutant-degrading microorganisms (Su et al. 2013). Enhancing production of *M. luteus* protein provides a new insight into bacterial degradation of pollutants and is helpful for efficacy testing of bioremediation.

## Conclusions

Plackett-Burman design and central composite design were employed to rapidly identify significant variables as well as to optimize medium composition and culture conditions for protein production by *Micrococcus luteus.* The results indicated that maximum protein production could be achieved when the significant variables of mineral solution, Lithium L-lactate, initial pH and incubation time were set at 1.5 ml/L, 8.75 g/L, 7.5 and 48 h, respectively. The predicated values calculated with the model coincided with the experimental values. Protein concentration in the *M. luteus* culture supernatant was obviously increased with optimization, which fitted well with the observed fluorescence intensity. The results confirmed the validity and practicability of this statistical optimization strategy.

## Methods

### Microorganisms and cultivation

*Micrococcus luteus* IAM 14879 (=JCM 21373 = NCIMB 13267) (Ding 2004; Mukamolova et al. 1998) was used in the present study. Cultures were grown in lactate minimal medium (LMM) containing (per liter): 4.0 g NH_4_Cl, 1.4 g KH_2_PO_4_, 0.005 g Biotin, 0.02 g L-Methionine, 0.04 g Thiamine B1, 1.0 g Inosine, 0.03 g MgSO_4_, 10.0 g Lithium L-lactate, and 1.0 mL of trace element solution, adjusted to pH 7.5. The trace element solution contained (per liter): 0.375 g CuSO_4_ · 5H_2_O, 0.785 g MnCl_2_ · 4H_2_O, 0.183 g FeSO_4_ · 7H_2_O, 0.029 g Na_2_MoO_4_ · 2H_2_O, and 0.089 g ZnSO_4_ · 7H_2_O. Seed cultures were prepared by transferring a pure culture into a 250 mL flask containing 80 mL of LMM medium and inoculating at 30°C on a rotary shaker at 160 rpm for 36 h. A portion of the seed culture was used to inoculate LMM medium (4%, v/v) which was cultured as previously described until the cells reached stationary phase. For the optimization experiments, medium composition and culture conditions were varied according to experimental design.

### Determination of protein yield

The resultant culture was centrifuged (8000 rpm, 15 min) to separate the cells, the centrifugal supernatant filtered through a 0.22 μm filter to remove floating cells and the supernatant assayed for protein yield. Protein content was measured using a modified Bradford Protein Assay Kit (Sangon Biotech, Shanghai, China) and plate read at 595 nm in a microplate reader (Thermo Scientific, Rockford, USA) (Ma et al. 2013). A standard curve was created using bovine serum albumin (BSA) protein, and protein concentration of samples was determined by comparison to the standard curve.

### Experimental design and statistical analysis

#### *Plackett-Burman design*

Plackett-Burman design is an effective technique for screening and evaluating significant factors that influence a response. The technique is based on the following first-order polynomial model:2

where *Y* represents the response (protein yield), *β*_0_ is the model intercept, *β*_*i*_ is the linear coefficient, *χ*_i_ is the level of the independent variable , and *k* represents the number of variables (Feng et al. 2010; Liu et al. 2010).

Seven medium components (NH_4_Cl, KH_2_PO_4_, L-Methionine, MgSO_4_, Inosine, mineral solution and Lithium L-lactate) and four culture conditions (initial pH, incubation time, incubation temperature and inoculum size) were investigated to determine the significant variables affecting protein production. Based on PBD, each variable was prepared at two levels -1 for low level and +1 for high level. Table 2 showed the levels of each variable and the 13 variables were investigated in 20 experimental runs (Table 1). All the runs were performed in triplicate and the average value was used as the response. The variables significant at a 95% level (*P* < 0.05) were deemed to have a significant effect on protein production, and were evaluated in further optimization experiments.

#### *Central composite design*

Based on the results of PBD, the significant variables of mineral solution (X_3_), Lithium L-lactate (X_4_), initial pH (X_8_) and incubation time (X_9_) were further optimized using Central composite design. A 2^4^ CCD with six replicates at the central point leading to 30 experiments was employed to optimize the conditions for improving protein yield. For statistical calculations, the relationship between the coded values and actual values are described by the following equation:3

where *χ*_*i*_ and *Α*_*i*_ are the coded value and actual value of the independent variable, respectively, *Α*_0_ is the actual value of the *Α*_*i*_ at the central point, and *ΔΑ*_*i*_ is the step change (Cao et al. 2010). According to the dependent variable of protein yield, each of the four variables was evaluated at five coded levels (-2, -1, 0, +1 and +2) and the final values were shown in Table 3.

For predicting the optimal point, a second-order quadratic equation describing the relationship between independent variable and response was developed. Design-based experimental data is used to fit a second-order polynomial equation:4

where Y is the predicted response, *β*_0_ is the interception coefficient, *β*_*i*_ is the linear coefficient, *β*_*ii*_ is the quadratic coefficient, and *β*_*ij*_ is the interactive coefficient (Körbahti and Rauf 2009; Mizumoto and Shoda 2007).

#### *Statistical analysis*

Design-Expert software (Version 8.0.5.0, Stat-Ease Inc., Minneapolis, USA) was used for regression and graphical analyses of the data. All experiments were performed in triplicate and the average of means was calculated. SPSS software (version 18.0) was used for data analysis and statistical analysis of the standard deviation (SD) to determine statistical significance.

### Validation of the models and the optimized conditions

The validation experiments were conducted under the optimized medium composition and culture conditions. By comparing the actual and predicted values, the validation of the models was investigated. In addition, the change of protein concentration in *M. luteus* culture supernatant with and without optimization was measured. And then *M. luteus* culture supernatant was analyzed by monitoring changed induced by optimization in synchronous fluorescence and three-dimensional fluorescence spectra. All fluorescence spectra were recorded on a Fluoromax-4 spectrometer (Horiba Co., Ltd. Kyoto, Japan) equipped with 1.0 cm quartz cells and a thermostat bath. *M. luteus* culture supernatant fluorescence emission spectra (λ_ex_ 280 nm, λ_em_ 300-450 nm) were measured with excitation and emission slits both at 5 nm (Keeratiurai et al. 2012). The three-dimensional fluorescence spectrum was performed according to the modified method by Jiang et al. (Jiang et al. 2013): the emission and excitation wavelengths were at 275-550 nm and 250-500 nm, respectively. The widths of the emission slit and excitation slit were set to 2.0 and 4.0 nm, respectively.
